# Green Recovery: Europe and the World

**DOI:** 10.1007/s10272-020-0933-x

**Published:** 2020-12-01

**Authors:** Robert Pollin

**Affiliations:** Department of Economics, University of Massachusetts Amherst, 412 North Pleasant Street, MA 01002 Amherst, USA

## Abstract

In Europe, and elsewhere, it is critical to recognize that this clean energy investment project will pay for itself in full over time in strictly financial terms – i.e. in addition to its obvious ecological benefits.

The October 2018 report of the Intergovernmental Panel on Climate Change (IPCC), the most authoritative global organization advancing climate change research, presented an unequivocal case for urgent action. The report emphasized the necessity of limiting the increase in global mean temperatures to 1.5 degrees Celsius (1.50C) above pre-industrial levels as of 2100. This will substantially reduce the risks of heat extremes, heavy precipitation, droughts, sea level rise, biodiversity losses and corresponding impacts on health, livelihoods, food security, water supply and human security. The IPCC concludes that to achieve the 1.50C maximum global mean temperature increase target by 2100, global net carbon dioxide ($CO_2$) emissions will have to fall by about 45% as of 2030 and reach net zero emissions by 2050 (IPCC, 2018).

## Basics of a global and European Green New Deal

In my view, the core of the global Green New Deal is to advance a global project to hit these IPCC targets, and to accomplish this in a way that also expands decent job opportunities and raises mass living standards for working people and the poor throughout the world. The overall project must also include just transition policies for workers and communities whose livelihoods now depend on the fossil fuel industries, and will therefore experience job losses and economic disruptions as these industries necessarily contract.

These features of a global Green New Deal are in full alignment with the basic premises of the European Green Deal – what European Commission (EC) documents have called its "man on the moon" project. As described in the EC program for a "Climate Neutral Europe by 2050," the stated objectives of the European Green Deal include achieving a 55% reduction in all greenhouse gas emissions by 2030 and to reach net zero emissions by 2050. The European Green Deal also emphasizes just transition measures for workers and communities that are currently dependent on the fossil fuel industry. The EC program describes its project as follows:

> The strategy shows how Europe can lead the way to climate neutrality by investing into realistic technological solutions, empowering citizens, and aligning action in key areas such as industrial policy, finance, or research – while ensuring social fairness for a just transition (European Commission, 2019a).

In fact, as a purely analytic proposition – independent of the myriad of political and economic forces arrayed around these matters – it is entirely realistic to allow that global $CO_2$ emissions can be driven to net zero by 2050. By my higher-end estimate, it will require an average level of investment spending throughout the global economy of about 2.5% of global GDP per year, focused in two areas: 1) dramatically improving energy efficiency standards in the stock of buildings, automobiles and public transportation systems, and industrial production processes; and 2) equally dramatically expanding the supply of clean renewable energy sources – primarily solar and wind power – to deliver electricity at competitive prices relative to fossil fuels and nuclear power to all sectors and in all regions of the globe. Clean energy investments will also need to be complemented in other areas, the most important of which is to stop deforestation and support afforestation.[1]

Focusing on the clean energy transformation, the level of necessary investments would amount to about $2.5 trillion in the first year of the program, which we assume here would be 2021. Spending would then average about $4.5 trillion per year between 2021 and 2050. Total global clean energy investment spending for the full-scale 30-year investment cycle 2021-2050 would amount to about $120 trillion.

These figures are for overall investment spending, including from both the public and private sectors. Establishing the right mix between public and private investment will be a major consideration within the industrial and financing policies framework. On a global scale, it is reasonable to assume that clean energy investments should be divided roughly equally – i.e. 50% public and 50% private investment respectively.

For Europe, it is likely that the public sector share of overall investment could be lower, at perhaps 25%. This is because private clean energy investment activity is already relatively well advanced in Europe, including investments in small-scale cooperative clean energy enterprises (Pollin, 2015, 92-109). Still, in Europe and elsewhere, a major part of the policy challenge will be to determine how to leverage the public interventions most effectively to create strong incentives for private investors, large and small, while also maintaining tight regulations over their activities. These efforts would operationalize the perspective articulated in September 2020 by Isabel Schnabel, a member of the European Central Bank Executive Board, that "Collective action, by governments, firms, investors, households, and central banks, including the European Central Bank, is required to accelerate the transition towards a carbon-neutral economy" (Arnold, 2020).

In Europe, and elsewhere, it is critical to recognize that this clean energy investment project will pay for itself in full over time in strictly financial terms – i.e. in addition to its obvious ecological benefits. More specifically, it will deliver lower energy costs for energy consumers in all regions of the world. This results because raising energy efficiency standards means that, by definition, consumers will spend less for a given amount of energy services through, for example, installing high-efficiency heat pumps in buildings to replace low-efficiency conventional heating, ventilation and cooling equipment. Moreover, the costs of supplying energy through solar and wind power, as well as geothermal and hydro sources, are now, on average, roughly equal to or lower than those for fossil fuels and nuclear energy. Specifically, as of 2019, the average global cost of electricity from utility-scale renewable energy sources was 6.6 cents (in US dollars) for solar, 5.3 cents for onshore wind, 4.2 cents for hydro and 7.3 cents for geothermal. The range for fossil fuel sources of electricity was between 5.0 and 17.7 cents (International Renewable Energy Agency, 2019). As a result, the initial upfront investment outlays in high efficiency and renewables can be repaid over time through the cost savings that will be forthcoming.

The consumption of oil, coal and natural gas will need to fall to zero or near-zero over this same 30-year period. The rate of decline can begin at a relatively modest 3.5% in the initial years of the transition program, but will then increase every year in percentage terms, as the base level of fossil fuel supply contracts annually through 2050. Of course, both the privately owned fossil fuel companies, such as Exxon-Mobil and Chevron, and equally, the publicly owned companies such as Saudi Aramco and Gazprom in Russia, have massive self-interests at stake in preventing reductions in fossil fuel consumption as well as enormous political power. These powerful vested interests will simply have to be defeated.

It is, finally, critical to recognize that these clean energy investments will be a major source of job creation. In fact, investing money in *anything* creates jobs. The relevant questions for assessing the job impacts of the clean energy program are therefore: 1) *How many* jobs will get

1 Discussions on these points are in Chomsky and Pollin (2020) and more fully Pollin (2020).

created over time through building a green economy and, correspondingly, how many jobs will be lost through the phase-out of the fossil fuel industry?; and 2) *How quickly* can jobs be generated under a clean energy investment program? If jobs can be created quickly, then the clean energy investment program can serve as a major engine of short-term recovery from the COVID-19 recession as well as a long-term framework for supporting both rising mass living standards and ecological sustainability.

## Job creation in Europe through clean energy investments

Table 1 reports estimates that I have generated with co-authors on job creation in Germany, Spain and Greece.[2] These figures are derived on the basis of the national input-output tables for these three countries. Row 1 reports on employment/output ratios for the respective economies for clean energy investments – i.e. total jobs created per €1 billion in investment spending in energy efficiency and renewable energy. As we see, the figure for Germany is roughly 11,000 jobs per €1 billion in expenditures. In Spain and Greece, the figures are much higher, and nearly identical, at 23,000 jobs per €1 billion in spending in Spain and 22,000 jobs per €1 billion in Greece. The figures for Spain and Greece are roughly double that for Germany because average wages in the relevant sectors in Spain and Greece are roughly half those in Germany.

Rows 2 and 3 report on the level of job creation when each country invests 2.5% of GDP in clean energy (using 2019 GDP figures), and how much that represents as a share of the country's overall labor force. As we see, in Germany, about 1.4 million jobs would be generated, equal to about 2.2% of the country's 2019 total workforce. For Spain, the job expansion would be almost 700,000 jobs, amounting to about 3.0% of the 2019 labor force. In Greece, the level of job creation, at about 100,000 jobs, represents an increase, similar to Germany, of about 2.2% of its 2019 labor force.

On its own, this level of job creation would not be sufficient to produce a full recovery from the COVID-19 recession in any of the three countries. As we see in row 4 of the table, according to the most recent figures, unemployment stands as of this writing at 6.3% for Germany. Thus, for Germany, all else equal, clean energy investments at 2.5% of GDP would be able to reduce unemployment to 4.1%. For Spain and Greece, current unemployment rates are much higher, at 16.2% and 17.3%

Table 1
**Job creation in Germany, Spain and Greece through energy efficiency and clean renewable energy investments**

| | Germany | Spain | Greece |
|---|---|---|---|
| 1) Job creation per €1 billion investments (rounded) | 11,000 | 23,000 | 22,000 |
| 2) Job creation by investments at 2.5% of GDP (rounded) | 1.4 million | 690,000 | 100,000 |
| 3) Job creation as share of 2019 labor force | 2.2% | 3.0% | 2.2% |
| 4) Most recent official unemployment figures | 6.3% | 16.2% | 17.3% |

Notes: Investments at 2.5% of 2019 GDP; Figures include direct, indirect and induced jobs.

Sources: Row 1 figures are from Pollin et al. (2015a) for Germany, Pollin et al. (2015b) for Spain, and Pollin (2019) for Greece. Rows 2-4 figures are from the Federal Reserve Bank of St. Louis FRED database.

respectively. The roughly 2%-3% reduction in the unemployment rate generated by clean energy investments at 2.5% of GDP will certainly be beneficial. But for both countries, this level of job expansion will need to be one component of a larger set of initiatives to achieve a robust short-term recovery.[3]

Part of the solution should be to increase the amounts of clean energy investments beyond the average annual figure of 2.5% of GDP that will be needed over a 30-year period. This increased investment level is justifiable both in terms of its short-run impact on jobs, but equally with respect to the climate stabilization project itself. This is because, to achieve the IPCC's intermediate target of a 45% emissions reduction by 2030, or the EC's more ambitious 2030 target of a 55% emissions reduction, it will be necessary for clean energy investments between now and 2030 to be greater than the overall 2021-2050 average of 2.5% of GDP. This follows from the fact that there remain only nine years, until 2030, to bring emissions down by roughly 50% from current levels, while there would still be 20 additional years – from 2031 to 2050, i.e. twice as much time – to reduce emissions from the 2030 level to net zero.

Another important factor in assessing job impacts of a clean energy transition is that Germany, Greece and Spain are all net fossil fuel importing countries, as are

2 See Pollin et al. (2015a) for Germany, (2015b) for Spain and Pollin (2019) for Greece.

3 Unemployment figures for all three countries are the most recent unadjusted rates reported at the Federal Reserve Bank of St. Louis FRED website as of 11 November 2020.

most Western European economies.[4] As such, their fossil fuel production activities, along with their refining and marketing sectors, are small in comparison with a clean energy investment project at 2.5% or more of GDP. As such, on a net basis, these economies will be able to reduce their energy import bills as their domestic clean energy sectors become capable of providing an increasing share of their economy's overall energy supply. Correspondingly, this import substitution effect will become an increasing source of job creation in all three countries, along with all other net fossil fuel importing economies. For the case of Greece, the annual net energy import bill averaged 2.8% of GDP between 2002 and 2018. Thus, to be able to eliminate that extent of import dependency over time will produce a level of job expansion roughly equivalent to the injection of 2.5% of GDP per year through clean energy investments – i.e. roughly 100,000 jobs per year (Pollin, 2019).

## Bringing the European Green Deal to scale

The level of spending that is being budgeted to date for the European Green Deal is well below what will be required for the European Union to achieve its stated emission reduction targets. To date, according to its main official documents, the EC estimates a budget of €1 trillion over 2021-2030 for everything, including clean energy investments as well as just transition programs (European Commission, 2020). This includes funding from all public and private sources, with about half of the money coming from the EU budget, and the other half provided by a combination of national governments and private investments. This level of funding amounts to an average of about €100 billion per year in total spending over 2021-2030, being equal to only about 0.7% of total EU GDP per year.[5]

To date, the EU is more advanced than the rest of the world in moving onto a clean energy investment growth path. We could therefore allow that the EU countries could achieve their 2030 and 2050 emission reduction targets with investment levels below the 2.5% of GDP global average, at perhaps 2% of GDP per year. But even this lower target would still imply clean energy investments within the EU at roughly €300 billion per year between 2021 and 2030, i.e. three times what the EC is proposing as their total Green Deal spending commitment. Indeed, other official EC documents state that the budgetary requirements for meeting its 2030 emission reduction goals would have to be closer to 1.5% of GDP, i.e. more than €200 billion per year (European Commission, 2019b). This lack of clarity within the EC itself underscores the need for European policymakers and the public to focus on the scale of the project required to achieve its climate stabilization goals.

Nevertheless, it still remains a major initial breakthrough that the European Union has committed itself, at least on paper, to achieving, and even exceeding, the IPCC's emission reduction targets. It is now crucial for European policymakers and, more so, the European public to recognize that: 1) Europe will not have to sacrifice jobs and living standards to hit what are now both the EU's and IPCC's emission reduction targets; 2) Building a clean energy infrastructure will save consumers money over time; and 3) The clean energy investment project can serve both as an effective short-term economic recovery program as well as a long-term sustainable growth path.

4 https://ec.europa.eu/eurostat/statistics-explained/index.php/Energy_production_and_imports.

5 This assumes that EU GDP grows at an average rate of about 1.5% per year, the average rate at which the region grew between 1999 and 2019.
